# Complete Genome Sequence of *Erythrobacter* sp. Strain SDW2, a Marine Bacterium Producing a Yellow Xanthophyll Pigment

**DOI:** 10.1128/mra.00812-22

**Published:** 2022-11-14

**Authors:** Sun-Wook Jeong, Yong Jun Choi

**Affiliations:** a School of Environmental Engineering, University of Seoul, Seoul, Republic of Korea; Montana State University

## Abstract

*Erythrobacter* sp. strain SDW2, which is capable of biosynthesizing a yellow xanthophyll pigment, was isolated from coastal seawater. Here, we report the complete genome sequence of this marine bacterial strain, with a genome size of 2,920,893 bp (64.38% G+C content) and 2,859 genes.

## ANNOUNCEMENT

Marine bacteria have received considerable attention as an industrial platform microbial resource for the production of value-added biocompounds with potential for use in pharmaceuticals and dietary supplements (1, 2). Previously, we reported that the newly isolated marine bacterium *Erythrobacter* sp. strain SDW2 from coastal seawater in Jeju Island, Republic of Korea, can produce pure yellow xanthophyll pigment exhibiting remarkable antioxidation activity (3). In this study, whole-genome sequencing of *Erythrobacter* sp. strain SDW2 was performed, which may help in designing a systematic engineering strategy to achieve industrial xanthophyll pigment biosynthesis and large-scale production.

Strain SDW2, which was isolated as previously described (3), was kept at −80°C in marine broth (KisanBio, Seoul, Republic of Korea) supplemented with 20% glycerol prior to use and was deposited in the Korean Agricultural Culture Collection (KACC), National Institute of Agricultural Science, Republic of Korea (http://genebank.rda.go.kr) (accession no. KACC 81198BP). The genome of *Erythrobacter* sp. strain SDW2 was constructed *de novo* from PacBio sequencing data. Genome sequencing was performed by CJ Bioscience, Inc. (Seoul, Republic of Korea). Genomic DNA (gDNA) was extracted from cells that had been grown at 30°C in 20 mL of marine broth for 24 h and purified using a Wizard gDNA isolation kit (Promega, Madison, WI, USA). The gDNA (5 μg) was sheared using the Megaruptor3 G-tube and then purified to remove DNA fragments less than 3 kb with AMpure XP beads, according to the manufacturer’s recommendations.

The SMRTbell library was prepared by using a SMRTbell Express template prep kit (Pacific Bioscience, catalog no. 101-357-000) and sequenced on the Sequel sequencing platform using a Sequel sequencing kit (version 3.0) and a SMRT Cell 1M (version 2) device (Pacific Bioscience). Data collection was carried out through a 600-min movie per each SMRT Cell 1M device. All software was run with the default parameters unless otherwise specified. The sequencing reads were assembled with SMRT link version 10.1.0.119588 using the Microbial Assembly protocol (Pacific Bioscience). In total, 272,328 subreads (average subread length, 7,772 bp; subread *N*_50_, 9,803 bp) of strain SDW2 were generated. The genome was assembled into a single contig with no additional replicons. Circlator version 1.4.0 (Sanger Institute) with default parameter setting values (genomeSize = 2.9 Mb) was used to remove identified overlapping ends, circularize the genome, and rotate the origin of replication to the starting position of the *dnaA* gene (4). The total genome size was estimated at 2,920,893 bp with a G+C content of 64.38% (average depth of sequencing coverage of 689.0×).

The genome of SDW2 strain was annotated via the NCBI Prokaryotic Genome Annotation Pipeline (PGAP) version 5.3 (5), which identified 2,859 genes, 2,793 coding DNA sequences (CDSs), 3 rRNAs, 44 tRNAs, 3 noncoding RNAs (ncRNAs), and 16 pseudogenes. 16S rRNA gene sequence analysis using Ez-Taxon (6) revealed that strain SDW2 showed 98.2% similarity with Erythrobacter dokdonensis DSW-74^T^. Subsequently, the average nucleotide identity (ANI) (7) and digital DNA-DNA hybridization (dDDH) value (formula *d*_4_) (8) between the genomes of strain SDW2 and *E. dokdonensis* DSW-74^T^ (accession number GCF_001677335.1) were found to be 74.6% (coverage, 30.9%) and 20%, respectively, far below the cutoff points for the novel species (<95% ANI, <70% dDDH) (9). These results indicate that strain SDW2 belongs to a new species lineage of the genus *Erythrobacter*.

### Data availability.

The *Erythrobacter* sp. strain SDW2 genome sequence has been deposited in GenBank under accession number CP090370. The PacBio reads have been deposited in the Sequence Read Archive (SRA) under accession number SRX13594282. The BioSample and BioProject numbers are SAMN24505124 and PRJNA793158, respectively.
